# Dual Inhibition of Histone Deacetylases and the Mechanistic Target of Rapamycin Promotes Apoptosis in Cell Line Models of Uveal Melanoma

**DOI:** 10.1167/iovs.62.12.16

**Published:** 2021-09-17

**Authors:** Ruchi P. Patel, Joanna R. Thomas, Katherine M. Curt, Christina M. Fitzsimmons, Pedro J. Batista, Susan E. Bates, Michael M. Gottesman, Robert W. Robey

**Affiliations:** 1 Laboratory of Cell Biology, Center for Cancer Research, National Cancer Institute, National Institutes of Health, Bethesda, Maryland, United States; 2 Columbia University Medical Center, Division of Hematology/Oncology, New York, New York, United States

**Keywords:** uveal melanoma, histone-deacetylase inhibitor, romidepsin, mTOR inhibition

## Abstract

**Purpose:**

Over 90% of uveal melanomas harbor pathogenic variants of the *GNAQ* or *GNA11* genes that activate survival pathways. As previous studies found that Ras-mutated cell lines were vulnerable to a combination of survival pathway inhibitors and the histone-deacetylase inhibitor romidepsin, we investigated whether this combination would be effective in models of uveal melanoma.

**Methods:**

A small-scale screen of inhibitors of bromodomain-containing protein 4 (BRD4; OTX-015), extracellular signal-related kinase (ERK; ulixertinib), mechanistic target of rapamycin (mTOR; AZD-8055), or phosphoinositide 3-kinase (PI3K; GDC-0941) combined with a clinically relevant administration of romidepsin was performed on a panel of uveal melanoma cell lines (92.1, Mel202, MP38, and MP41) and apoptosis was quantified by flow cytometry after 48 hours. RNA sequencing analysis was performed on Mel202 cells treated with romidepsin alone, AZD-8055 alone, or the combination, and protein changes were validated by immunoblot.

**Results:**

AZD-8055 with romidepsin was the most effective combination in inducing apoptosis in the cell lines. Increased caspase-3 and PARP cleavage were noted in the cell lines when they were treated with romidepsin and mTOR inhibitors. RNA sequencing analysis of Mel202 cells revealed that apoptosis was the most affected pathway in the romidepsin/AZD-8055-treated cells. Increases in pro-apoptotic *BCL2L11* and decreases in anti-apoptotic *BIRC5* and *BCL2L1* transcripts noted in the sequencing analysis were confirmed at the protein level in Mel202 cells.

**Conclusions:**

Our data suggest that romidepsin in combination with mTOR inhibition could be an effective treatment strategy against uveal melanoma due in part to changes in apoptotic proteins.

Histone deacetylase inhibitors (HDIs) are antitumor agents that lead to histone acetylation and promote a more open DNA structure that can lead to increased gene expression and eventual apoptosis in tumor cells.^1^ Despite the remarkable success in T-cell lymphoma,^2^ clinical trials of HDIs in solid tumors both as single agents and in combination with other chemotherapeutic drugs have been largely ineffective.^3^ Romidepsin (also known as depsipeptide [Dp]) is a potent HDI that primarily inhibits class I histone deacetylases (HDACs). As with other HDIs, the clinical efficacy of romidepsin in hematological tumors has not been replicated in solid tumors, suggesting intrinsic resistance to romidepsin.

Previous studies undertaken to identify mechanisms of resistance to romidepsin found that selection with romidepsin in cancer cell line models results in overexpression of the ATP-binding cassette transporter ABCB1 (P-glycoprotein [P-gp])^4^^–^^7^; however, overexpression of *ABCB1* was not found in clinical samples obtained from patients with resistant disease.^8^ To characterize non-P-gp mechanisms of resistance to romidepsin, we selected the T-cell lymphoma cell line HuT78 with romidepsin in the presence of P-gp inhibitors to prevent overexpression of P-gp as a resistance mechanism. The resulting cell lines demonstrated activation of the mitogen-activated protein kinase (MAPK) pathway as a resistance mechanism,^9^ leading us to consider whether other genetic mutations that activate survival pathways might be made susceptible to romidepsin treatment when combined with specific inhibitors. To test this hypothesis, we applied inhibitors of the MAPK and phosphoinositide 3-kinase (PI3K) pathways in combination with romidepsin to Ras-mutated cell lines, as mutations in Ras are known to activate the MAPK and PI3K pathways. We found that these combinations uniquely sensitized Ras-mutated cell lines to romidepsin.^10^ Other groups have reported similar findings,^11^^,^^12^ thus providing additional support for the idea that activation of survival pathways provides intrinsic protection from HDI treatment. We further sought to investigate romidepsin sensitization in other cancers.

Uveal melanoma is the most common intraocular malignancy in adults, affecting approximately five to six individuals per million people in the United States.^13^ Nearly half of the patients diagnosed with primary uveal melanoma will develop metastatic disease, but there are currently no effective therapies for metastatic uveal melanoma.^14^ Interestingly, activation of the MAPK and PI3K survival pathways is observed in uveal melanoma; however, driver mutations in *BRAF* or *NRAS* commonly present in cutaneous melanoma are not observed in uveal melanoma.^15^ Instead, over 90% of uveal melanomas aberrantly activate G protein-coupled receptor signaling, specifically through mutually exclusive somatic pathogenic variants of the heterotrimeric G protein alpha subunits *GNAQ* and *GNA11*.^16^ Studies have found that these activating mutations stimulate phospholipase C to activate the MAPK pathway^17^ and inhibiting related pathways significantly reduces proliferation of *GNAQ*/*GNA11* mutant cells.^18^ The PI3K/AKT pathway, in contrast, is not activated due to mutations in *GNAQ/GNA11*, but rather by an upregulation of receptor tyrosine kinases or loss of the phosphatase and tensin homolog protein (PTEN).^17^ Interestingly, recent studies have shown that the mechanistic target of rapamycin (mTOR) signaling is activated in the absence of AKT phosphorylation. This suggests that mTOR stimulation could be a result of the MAPK cascade activation that arises from *GNAQ/GNA11* pathogenic variants.^19^ Finally, approximately 40% of uveal melanomas carry loss-of-function pathogenic variants of the *BAP1* tumor suppressor gene and these variants are associated with increased metastatic risk.^16^

This study examines the effect of various pathway inhibitors in combination with romidepsin on multiple uveal melanoma cell lines. In particular, we investigated the outcome of romidepsin treatment in combination with PI3K, mTOR, extracellular signal-regulated kinase (ERK), and bromodomain (BRD) inhibition. The PI3K and mTOR inhibitors were chosen to further examine the effects of inhibiting each pathway separately. As studies have also found the MAPK pathway to be activated by *GNAQ* and *GNA11* pathogenic variants, we chose to evaluate inhibition of ERK, a downstream target of this pathway. Studies have also suggested that BRD inhibitors have some effects similar to those of MEK inhibitors, such as upregulation of Bim and downregulation of c-Myc,^20^ leading us to combine romidepsin with BRD inhibition. We hypothesized that adding romidepsin to one of these pathway inhibitors could be an effective therapy against uveal melanoma tumors that harbor a mutation in *GNAQ/GNA11*.

## Materials and Methods

### Cell Culture

The uveal melanoma cell lines Mel202^21^ and 92.1^22^ were cultured in Roswell Park Memorial Institute medium 1640 (RPMI; Cat #11875-093; Gibco/Thermo Fisher Scientific, Waltham, MA, USA) supplemented with 10% fetal bovine serum (FBS; Cat #26140-079; Gibco/Thermo Fisher Scientific) and 1% penicillin and 1% streptomycin (Cat #15140-148; Gibco/Thermo Fisher Scientific) in a 37°C incubator with 5% CO_2_. MP41 and MP38^19^ were cultured in the same media with an additional 10% FBS. The 92.1 and Mel202 cell lines were purchased from MilliporeSigma (Burlington, MA, USA); MP41 and MP38 cell lines were purchased from American Type Culture Collection (ATCC; Manassas, VA, USA). The retinal pigmented epithelial line ARPE-19 was also obtained from ATCC and was maintained in DMEM:F12 medium with 10% FCS. Cell lines were validated by short tandem repeat (STR) analysis (perfomed on samples sent to ATCC), and the profiles were consistent with those reported for Mel202 (https://web.expasy.org/cellosaurus/CVCL_C301), 92.1 (https://web.expasy.org/cellosaurus/CVCL_8607), MP41 (https://web.expasy.org/cellosaurus/CVCL_4D12), and MP38 (https://web.expasy.org/cellosaurus/CVCL_4D11). A summary of mutations in the *GNAQ*, *GNA11*, and/or *BAP1* in the cell lines is provided in Supplementary Table S1. STR profiles are provided in Supplementary Table S2. The STR profiles for the 92.1 and Mel202 cell lines were found to match those previously reported by Jager et al.^23^ and Griewank et al.^24^

### Chemicals

The HDI romidepsin (Cat #S3020) and the ERK inhibitor ulixertinib (Cat #S7854) were purchased from Selleck Chemicals (Houston, TX, USA). The PI3K inhibitor GDC-0941 (Cat #CT-G0941) and mTOR inhibitors AZD-8055 (Cat #CT-A8055) and NVP-BEZ235 (Cat #CT-BEZ) were from ChemieTek (Indianapolis, IN, USA). The mTOR inhibitor rapamycin (Cat #1292) was obtained from Tocris/R&D Systems (Minneapolis, MN, USA). The BRD inhibitor OTX-015 (Cat #15947) was purchased from Cayman Chemicals (Ann Arbor, MI, USA). The pan-caspase inhibitor Q-VD-OPh (Cat #A1901) was obtained from ApexBio Technology (Houston, TX, USA).

### Flow Cytometry

All cell lines were trypsinized, plated in a 6-well plate, and allowed to attach overnight. Cells were then treated with the indicated treatments and both floating and adherent cells were harvested 48 hours after the start of treatment. Cells were then incubated with SYTOX Green (Cat #S7020; Invitrogen/Thermo Fisher Scientific) and allophycocyanin-conjugated annexin V (APC Annexin V; Cat #640941; BioLegend, San Diego, CA, USA) for at least 20 minutes in Annexin Binding Buffer (BioLegend) according to the manufacturer's instructions. A FACSCanto II Flow Cytometer (Cat #338960; BD Biosciences, San Jose, CA, USA) was then used to analyze the samples; a total of 10,000 events were collected for each sample. The percentage of annexin-positive cells was determined using FlowJo software (version 10.4.2; FlowJo, Ashland, OR, USA).

### Immunoblot Analysis

Uveal melanoma cell lines were plated in a 100 × 20 mm polystyrene plate, allowed to attach overnight, and subsequently treated with indicated treatments for 24 hours. Both floating and adherent cells were then harvested and re-suspended in lysis buffer (50 mM Tris-HCl pH 7.4, 150 mM NaCl, 1% NP-40, 1% protease and phosphatase inhibitor cocktail; Cat #A32959; Pierce/Thermo Fisher Scientific, Waltham, MA, USA), sonicated, and centrifuged to remove cell debris. The supernatant was reserved and protein was loaded into a 4% to 12% Bis-Tris NuPAGE gel (Cat #NP0321PK2; Invitrogen/Thermo Fisher Scientific, Waltham, MA, USA), subjected to electrophoresis and transferred to a 0.2 µm pore size nitrocellulose membrane. The resulting membrane was blocked in Odyssey PBS Blocking Buffer (Cat #927-40000; LI-COR, Lincoln, NE, USA) for 30 minutes at room temperature and subsequently incubated with one or more of the following primary antibodies overnight: β-actin (1:10000; Cat #3700S), cleaved PARP (1:500; Cat #9546S), cleaved caspase-3 (1:1000; Cat #9661S), phospho-S6 (1:1000; Cat #62016S), S6 (1:1000; Cat #2217S), Mcl-1 (1:1000; Cat #4572S), Bim (1:500; Cat #2933S), Bcl-x_L_ (1:1000; Cat #2762S), Bid (1:1000; Cat #8762S), Bax (1:1000; Cat #2772S), Bak (1:1000; Cat #3814S), Puma (1:1000; Cat #4976S), or Survivin (1:500; Cat #2808S). All antibodies were purchased from Cell Signaling Technology (Beverly, MA, USA). Membranes were washed with 0.5X TBS (Cat #351-086-131; Quality Biological, Gaithersburg, MD, USA) containing 0.5% TBS-Tween-20 (Cat #IBB-181; Boston BioProducts, Worcester, MA, USA) 3 times for 5 minutes prior to and after incubation with IRDye anti-Rabbit (Cat# 926-32213; LI-COR, Lincoln, NE, USA) or anti-Mouse (Cat #926-68070; LI-COR) secondary antibody for 1 hour. Proteins were visualized using an Odyssey CLx imaging system (LI-COR).

### RNA Isolation and Sequencing

Mel202 cells were treated with 1 µM AZD-8055, 25 ng/mL romidepsin, or a combination of both drugs for 6 hours. Using an RNeasy Plus Mini Kit (Qiagen; Cat #74136), cells were then harvested and total RNA was isolated at room temperature according to the kit instructions. RNA sample integrity and quantity for three biological replicates were assessed by the Center for Cancer Research Genomics Core (CCR Genomics Core, Bethesda, MD, USA) using RNA ScreenTape Analysis (Cat #5067-5576; Agilent, Santa Clara, CA, USA). All RNA samples had RNA Integrity Numbers of 10.0. RNA sequencing was performed by the CCR Sequencing Facility and Genomics Technology Core (Frederick, MD, USA) on a NextSeq 500 Sequencing System (Illumina, San Diego, CA, USA) as 76 base paired-end reads using a TruSeq Stranded mRNA Library Prep (Illumina). Base calling was performed using Real-Time Analysis software (RTA; version 1.18.64; Illumina). Pre-alignment quality check determined that more than 93% of bases had a Phred-like quality score (Q-score; measure of base calling quality) above Q30. Reads of the samples were trimmed for adapters and low-quality bases using Cutadapt software (version 1.18)^25^ before alignment with the hg19 reference genome and the annotated transcripts using Spliced Transcripts Alignment to a Reference (STAR; version 2.6.1).^26^ Gene expression quantification analysis was performed for all samples using STAR and RNA-Seq by Expectation Maximization (RSEM; version 1.2.3.1)^27^ tools. Differential expression analyses between samples was computed using DESeq2 (version 1.22.2)^28^ within R Studio (version 1.1.456; Boston, MA, USA). Pathway analysis was conducted through iPathwayGuide (Advaita Bioinformatics; Plymouth, MI, USA).^29^ RNA Seq data were deposited in the Gene Expression Omnibus database (accession GSE155452).

### Statistical Analyses

GraphPad Prism 8 was used to determine all statistical analyses in this study, including standard deviation and statistical significance. Differences were considered statistically significant when *P* < 0.05 by a two-tailed Student's *t*-test.

## Results

### Preliminary Screening of Pathway Inhibitors Combined With Romidepsin

We chose four uveal melanoma cell lines with a diverse genetic background for our initial screen (see Supplementary Table S1). Two widely used cell lines in the field are 92.1 and Mel202, both of which harbor *GNAQ* pathogenic variants, but express the BAP1 protein. The two other cell lines tested in this study, MP38 and MP41, were established to better represent the genetic scope of uveal melanoma.^19^ The MP38 line harbors a mutant *GNAQ*; however, it also carries a *BAP1* pathogenic variant and does not express the corresponding BAP1 protein. MP41, in contrast, harbors a *GNA11* mutation but does express the BAP1 protein.

The cell lines were exposed to a variety of small-molecule targeted therapies (listed in Supplementary Table S3) OTX-015 (BRD4 inhibitor), ulixertinib (ERK inhibitor), GDC-0941 (PI3K inhibitor), and AZD-8055 (mTOR inhibitor—alone and in combination with romidepsin) and cell death was measured by flow cytometry. As romidepsin is administered as a 4-hour infusion and has a short half-life,^30^^–^^32^ we administered romidepsin as previously described so as to more closely simulate its clinical dosing regimen.^10^^,^^33^ Cells were treated with romidepsin (25 ng/mL) for 6 hours alone or in combination with small-molecule inhibitors, after which the medium was removed and romidepsin-free medium was added. Cells were then incubated for another 42 hours continuing with or without the small-molecule inhibitors. They were then stained with annexin V and SYTOX green and analyzed by flow cytometry.

**Figure 1.**
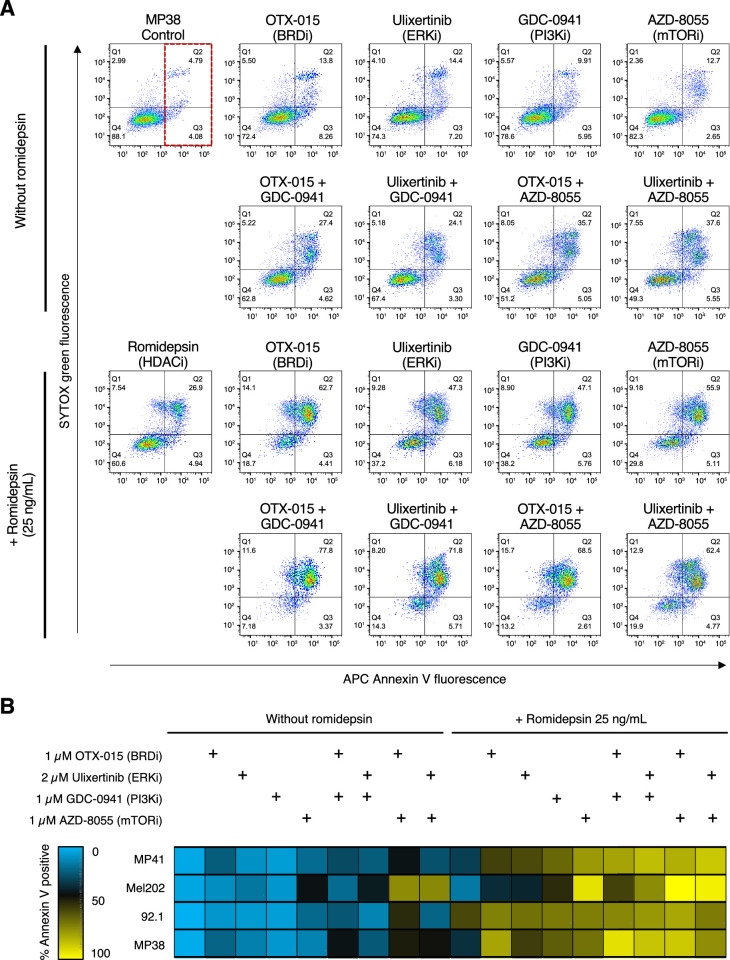
Preliminary screen for drug combinations with romidepsin that promote cell death in uveal melanoma cell lines. (**A**) MP38 cells were initially treated for 6 hours without or with 25 ng/mL romidepsin alone or in combination with pathway inhibitor(s) as indicated. Cells were subsequently incubated in romidepsin-free media in the absence or presence of pathway inhibitors for an additional 42 hours, stained with annexin V and SYTOX green, and analyzed by flow cytometry. Right-most quadrants of each (as indicated by sample portion of first graph boxed in *red*) represent annexin-positive cells. (**B**) Heat map containing data for annexin-positive cells for each treatment applied to MP41, Mel202, 92.1, and MP38 cells. The data shown represent the mean of three biological replicates.

Results from a single experiment with the MP38 cell line are displayed in Figure 1A. The right-most quadrant of each graph, boxed in red in the first graph in Figure 1A, represents the cells that were considered to be annexin-positive and were used to generate the heat map for all four cell lines shown in Figure 1B. Raw data used to generate the heat map can be found in Supplementary Table S4. As can be observed, treatment with pathway inhibitors as single agents did not result in increased apoptosis compared to untreated cells. Interestingly, combinations of the pathway inhibitors resulted in increased apoptosis, particularly when the BRD4 inhibitor OTX-015 was combined with the mTOR inhibitor AZD-8055. Short-term romidepsin treatment resulted in increased apoptosis predominantly in the 92.1 cell line, but the combination of romidepsin with the small molecule inhibitors yielded higher levels of apoptosis in all cell lines as compared to romidepsin alone. Due to its high degree of efficacy in all of the four uveal melanoma cell line models, we chose to further examine the combination of romidepsin with mTOR inhibitors in the cell line models.

### Combining Romidepsin With mTOR Inhibitors Promotes Cell Death in Uveal Melanoma Models

**Figure 2.**
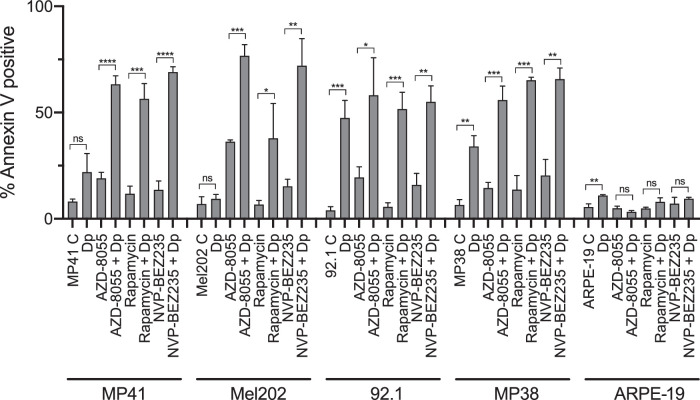
The mTOR inhibitors combined with romidepsin are effective in uveal melanoma cell line models. MP41, Mel202, 92.1, MP38, and ARPE-19 cells were treated for 6 hours with 25 ng/mL romidepsin alone or in combination with 1 µM of rapamycin or NVP-BEZ235 as indicated. Cells were then incubated in romidepsin-free media in the absence or presence of the mTOR inhibitor for an additional 42 hours, stained with annexin V and SYTOX green, and analyzed by flow cytometry. A quantitative analysis of the data is shown. Each bar represents the mean of three biological replicates with the standard deviation indicated by error bars. *n* = 3, ****P < 0.0001, ***P < 0.001, **P < 0.01, *P < 0.05.

To confirm that results from the romidepsin/mTOR inhibitor combination were not unique to AZD-8055, we tested the combination of two additional mTOR inhibitors with romidepsin in all four cell lines and characterized cell death by flow cytometry analysis. Additionally, we treated the retinal pigmented epitheilial line ARPE-19 with the combinations to determine their effects on normal epithelial tissue. Figure 2 shows the effects of 1 µM rapamycin and 1 µM NVP-BEZ235 in the absence and presence of a short-term romidepsin treatment. The mTOR inhibitors alone slightly increased apoptosis in some uveal melanoma cell lines; however, the combination of the mTOR inhibitors with romidepsin resulted in increased apoptosis in all uveal melanoma cell lines studied, except for the 92.1 cell line, which was highly sensitive to short-term romidepsin treatment alone. When tested against ARPE-19 cells, none of the inhibitors, alone or in combination, elicited appreciable annexin staining, suggesting limited toxicity on normal cells.

**Figure 3.**
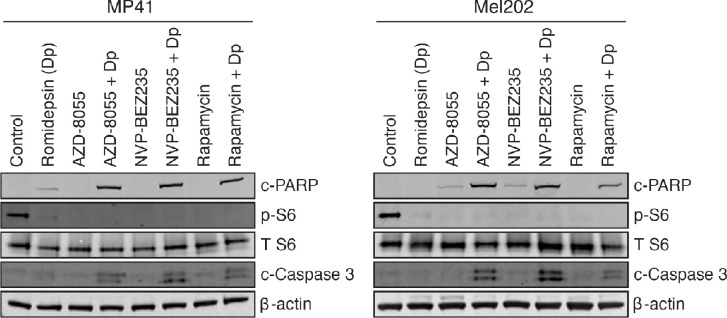
Combination of mTOR inhibitors with romidepsin leads to increased PARP and caspase cleavage. MP41 and Mel202 cells were treated for 6 hours with 25 ng/mL romidepsin alone or in combination with various mTOR inhibitors (1 µM AZD-8055, 1 µM NVP-BEZ235, or 1 µM rapamycin) as indicated. Cells were subsequently incubated in romidepsin-free media in the absence or presence of the mTOR inhibitor for an additional 18 hours, at which point cells were harvested. Cell lysates were then prepared, subjected to SDS-PAGE, and transferred to nitrocellulose membranes. Membranes were probed with one or more of the following antibodies: cleaved PARP (c-PARP), cleaved caspase-3 (c-Caspase 3), phospho-S6 (p-S6), or total S6 (T S6). β-actin served as a loading control. Each immunoblot was performed in at least two independent experiments.

We further validated the efficacy of combining romidepsin with mTOR inhibitors in the uveal melanoma cell lines by examining the downstream mTOR pathway target S6, as well as PARP and caspase-3 cleavage after treatment with the various mTOR inhibitors in the absence or presence of romidepsin. MP41 and Mel202 cells (Fig. 3) as well as 92.1 and MP38 cells (Supplementary Fig. S2) were treated with three mTOR inhibitors (AZD-8055, NVP-BEZ235, and rapamycin) for 24 hours, both without and with a short term 6-hour romidepsin treatment. As seen in Figure 3 and Supplementary Figure S2, a 24-hour treatment of all three mTOR inhibitors led to inhibition of the mTOR pathway, as demonstrated by a loss of S6 protein phosphorylation. Additionally, treatment with the mTOR inhibitors alone was found to be relatively nontoxic to uveal melanoma cells, as no cleaved PARP or caspase-3 was observed. However, with the addition of romidepsin, we observed increased PARP and caspase-3 cleavage, established markers of apoptosis. These results are in agreement with the data obtained by flow cytometry (see Fig. 2). Taken together, this demonstrates that the combination of romidepsin, administered in a clinically relevant manner, with an mTOR inhibitor increases cell death in uveal melanoma cell line models.

### RNA Sequencing Analysis Reveals Gene Changes in the Apoptosis Pathway

**Figure 4.**
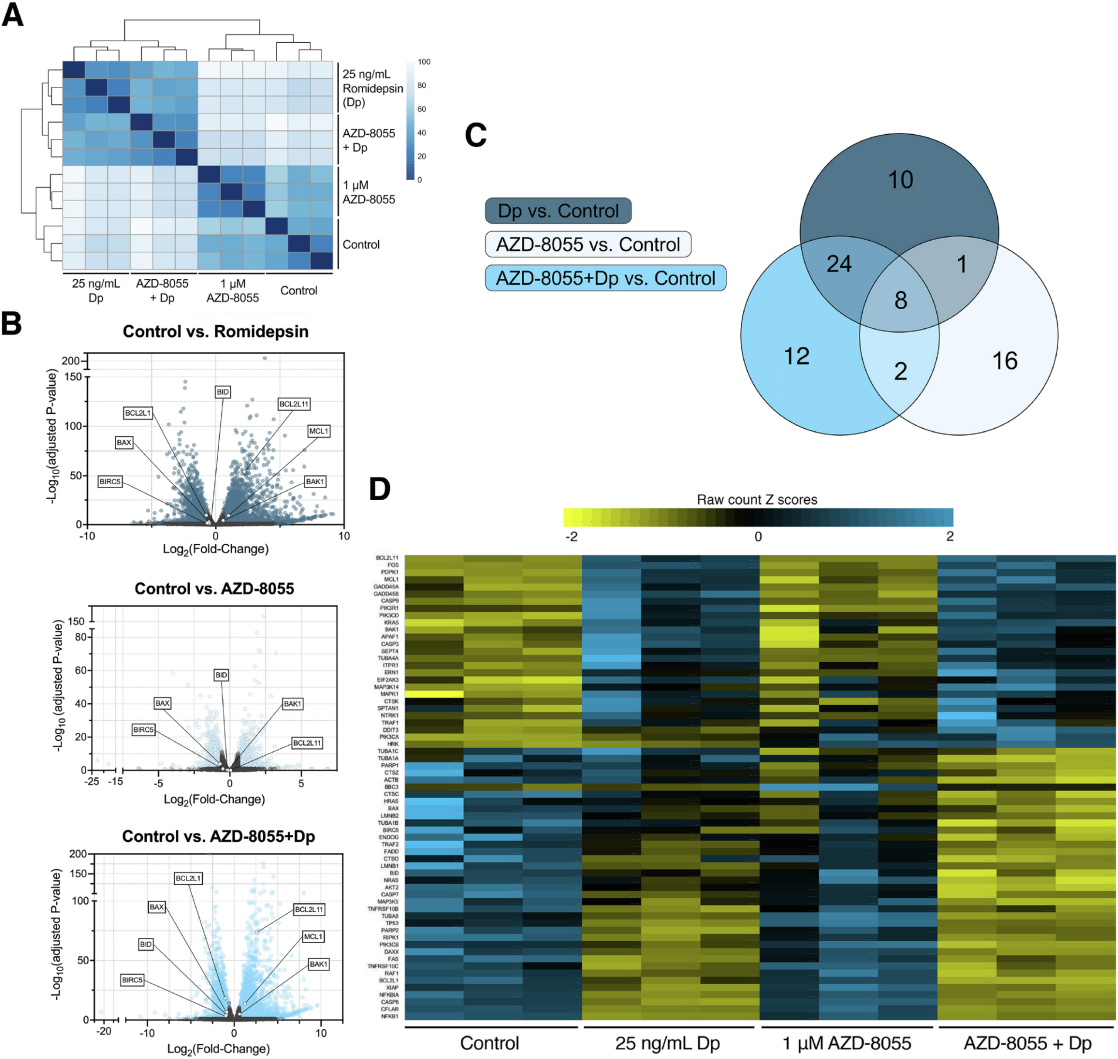
RNA sequencing analysis of Mel202 cells. Mel202 cells were left untreated or were treated with 1 µM of AZD-8055, 25 ng/mL romidepsin, or a combination of both for 6 hours. Total RNA was isolated from harvested cells and RNA sequencing was performed to analyze for differential gene expression after treatments. (**A**) Heat map displaying hierarchical clustering of samples based on sample-to-sample distances. (**B**) Differential gene expression of RNA sequencing analysis for each treatment scheme displayed as –log_10_ (adjusted *P* value) against the log_2_ (fold-change). Significant (adjusted *P* value < 0.05) genes with a log_2_ (fold-change) greater than two are displayed for Romidepsin [Dp] vs. control (top plot), AZD-8055 vs. control (middle plot), and AZD-8055 + Dp vs. control (bottom plot). Apoptotic genes that were probed via Western blot in Figure 5 are labeled here: Mcl-1, Bim (*BCL2L11*), Bcl-x_L_ (*BCL2L1*), Bid, Bak (*BAK1*), Bax, or Survivin (*BIRC5*). (**C**) Meta-analysis of RNA sequencing data using iPathwayGuide (www.advaitabio.com) determined 12 significant pathways specific to the AZD-8055 + Dp combination therapy treatment group. (**D**) Heat map of Z-scores of apoptotic genes analyzed by iPathwayGuide. The z-scores were calculated from raw read counts in the four treatment groups as indicated.

To analyze gene transcriptional changes that might contribute to toxicity of romidepsin/mTOR inhibitor combinations, we performed RNA sequencing analysis of the uveal melanoma cell line Mel202. Cells were treated with AZD-8055 (mTOR inhibitor), romidepsin, or a combination of both of these drugs for 6 hours, at which point we harvested cells, isolated total RNA, and subsequently performed RNA sequencing. Hierarchical clustering of the gene expression of the different treatment groups demonstrated that the gene expression of untreated cells was similar to that of cells treated with AZD-8055. In addition, the transcriptional changes observed in cells treated with romidepsin were most similar to changes seen in cells treated with both AZD-8055 and romidepsin (Fig. 4A). We compared the gene expression of each treatment group with the untreated control to observe which genes were upregulated and downregulated at the end of a 6-hour treatment (Fig. 4B).

We then analyzed RNA sequencing data using iPathway Guide (iPG, www.advaitabio.com) to visualize affected pathways post-treatment (Fig. 4C). The iPG uses a unique, combinatorial approach to identify significantly impacted pathways based on the over-representation of differentially expressed genes in a given pathway and the perturbation of that pathway.^29^^,^^34^^,^^35^ Using this analysis software, we compared each of the treatments (romidpesin alone, AZD-8055 alone, or the combination of romidepsin and AZD-8055) to untreated cells to determine if any pathways were altered by the treatments. Although no significant pathway enrichment was observed with the single drug treatments, we discovered 12 pathways that were significantly enriched by the combination therapy (AZD-8055 and romidepsin) compared to control cells (Table). Of these 12 pathways, the pathway most affected was apoptosis (*P* = 0.0008944). We next evaluated the expression of the genes that iPG includes in the apoptosis pathway in all four of our treatment groups. We calculated the z-score of each replicate per treatment for every gene used by iPG to establish apoptosis as specifically significant to the combination treatment group (Fig. 4D). Portraying the gene expression as z-scores in a heatmap allows visualization of the genes that are downregulated or upregulated by each of the treatments. Interestingly, a clear distinction between the untreated control group on the left and the dual-treatment group on the right can be observed. The expression of the apoptotic genes follows the same general clustering depicted in Figure 4A. Cells treated with AZD-8055 display gene expression similar to that of untreated cells in the control group, whereas cells treated with romidepsin alone display gene expression comparable to that of cells treated with both AZD-8055 and romidepsin. Genes that are downregulated in the untreated cells and AZD-8055-treated cells appear to be upregulated in the romidepsin-treated and combination therapy treated cells. The same trend is seen with upregulated genes in the untreated and AZD-8055-treated cells, shifting to downregulated when treated with romidepsin alone or the combination therapy.

**Table.** Pathways Determined by iPathwayGuide to be Specifically Significant to AZD-8055 + Romidepsin (Dp) Combination Therapy

	AZD-8055	Romidepsin (Dp)	AZD-8055 +
Pathway Name	vs. Control	vs. Control	Dp vs. Control
	***P*** **value**		
**Apoptosis**	**0.292**	**0.191**	**8.944e-4**
Glioma	0.693	0.051	0.004
Melanoma	0.548	0.125	0.005
Viral carcinogenesis	0.185	0.108	0.006
p53 signaling pathway	0.293	0.065	0.007
AGE-RAGE signaling pathway in diabetic complications	0.265	0.090	0.009
Thyroid hormone signaling pathway	0.780	0.055	0.015
Influenza A	0.306	0.447	0.026
Thyroid cancer	0.563	0.062	0.029
Kaposi's sarcoma-associated herpesvirus infection	0.531	0.245	0.031
EGFR tyrosinase kinase inhibitor resistance	0.079	0.068	0.039
Apoptosis – multiple species	0.245	0.054	0.044

### Validation of Changes in Apoptotic Proteins After Combination Romidepsin/mTOR Inhibitor Treatment

**Figure 5.**
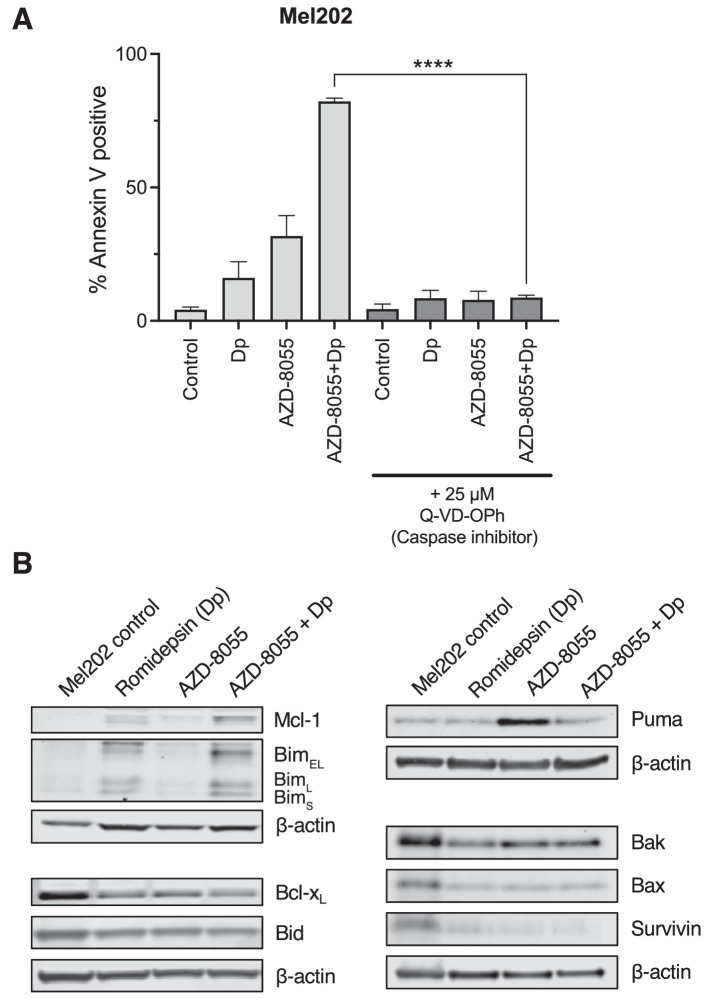
Apoptosis induced by combination of romidepsin and AZD-8055 is caspase-dependent. (**A**) Mel202 cells were treated for 6 hours with 25 ng/mL romidepsin alone or in combination with 1 µM AZD-8055 either in the presence or absence of 25 µM of the caspase inhibitor Q-VD-OPh. Cells were then incubated in romidepsin-free media in the absence or presence of AZD-8055 and Q-VD-OPh for an additional 42 hours, stained with annexin V and SYTOX green, and analyzed by flow cytometry. A quantitative analysis of the data is shown. Each bar represents the mean of three biological replicates with the standard deviation indicated by error bars (*n* = 3). (**B**) Mel202 cells were treated for 6 hours with 25 ng/mL romidepsin alone or in combination with 1 µM AZD-8055. Cells were subsequently incubated in romidepsin-free media in the absence or presence of AZD-8055 for an additional 18 hours, at which point cells were harvested. Cell lysates were then prepared, subjected to SDS-PAGE, and transferred to nitrocellulose membranes. Membranes were probed with one or more of the following antibodies: Mcl-1, Bim (with three splice variants Bim_EL_, Bim_L_, and Bim_S_), Bcl-x_L_, Bid, Puma, Bax, Bak, or Survivin. β-actin served as a loading control. At least two independent experiments were performed.

We and others have pointed to the importance of the intrinsic apoptosis pathway in the efficacy of HDIs, both as single agents and in drug combinations.^9^^,^^10^^,^^33^^,^^36^^–^^39^ The intrinsic apoptotic pathway, mediated by members of the BCL2 family of proteins, ultimately leads to release of cytochrome C from the mitochondria, triggering apoptosome formation and eventual cleavage of caspases.^40^ To determine if caspase cleavage is required for the apoptosis observed with the combination therapy, we treated Mel202 cells with 1 µM AZD-8055 alone or in combination with 25 ng/mL romidepsin as described above in the absence or presence of 25 µM Q-VD-OPh, a pan-caspase inhibitor. Cells were subsequently stained with annexin V and SYTOX green and analyzed by flow cytometry. In the absence of Q-VD-OPh, the average percentage of annexin-positive cells when treated with the combination therapy increased to 82% as compared to 19% and 32% when cells were treated with 25 ng/mL romidepsin or 1 µM AZD-8055 as individual treatments, respectively (Fig. 5A). In contrast, when Mel202 cells were treated with romidepsin and AZD-8055 in the presence of 25 µM Q-VD-OPh, apoptosis induced by all 3 treatments was significantly abrogated. Similar results were observed for 92.1, MP38, and MP41 cells (see Supplementary Fig. S2).

We confirmed the results of the RNA sequencing analysis by examining the effect of the romidepsin/AZD-8055 combination treatment on the expression of the BCL2 family proteins, including the pro-apoptotic proteins Bim and Puma, the anti-apoptotic proteins Mcl-1 and Bcl-x_L_, and the effector proteins Bak and Bax. We also examined the expression of Survivin, which has been shown to inhibit apoptosis via the intrinsic pathway (Fig. 5B). We found that mTOR inhibition alone did not increase expression of the pro-apoptotic protein Bim; however, short-term romidepsin treatment, alone or in combination with an mTOR inhibitor, resulted in increased Bim expression. The expression of the pro-apoptotic protein Bid was decreased by the romidepsin/mTOR inhibitor combination, whereas the expression of Puma was increased by mTOR inhibitor alone. Notably, the romidepsin/mTOR inhibition combination decreased the expression of the anti-apoptotic proteins Bcl-x_L_ and Survivin.

The expression of BCL2 family members was also examined in the 92.1, MP38, and MP41 cells treated with AZD-8044, romidepsin, or the combination (Supplementary Fig. S3). Expression of Bak and Bax did not change appreciably with any of the treatments. We found that romidepsin treatment led to increased MCL-1 expression in MP41 cells, similar to what was observed in the Mel202 line, but not in the other cell lines. Slight changes in BCL-XL expression were noted in the 92.1 and MP38 cell lines. Additionally, high levels of pro-apoptotic Bim were consistently observed in all cell lines tested when they were treated with romidepsin alone or in combination with AZD-8055 and appeared to be highest in cells treated with the romidepsin/AZD-8055 combination. This was especially true in the 92.1 cell line, where higher levels of the smaller Bim iosoforms were observed. The small Bim isoform (Bim_S_) is more effective at inducing apoptosis.^41^ We conclude that short-term romidepsin treatment with mTOR inhibition leads to changes in gene expression of apoptotic proteins, which may play a role in the observed cell death.

## Discussion

To date, the US Food and Drug Administration has approved only three HDIs as single agents for the treatment of cancer: vorinostat, romidepsin, and belinostat.^3^ All of these drugs have been approved for the treatment of T-cell lymphoma. However, the efficacy of HDIs as single agents against T-cell lymphoma has yet to be replicated for solid malignancies.^3^^,^^42^ This suggests that solid tumors harbor an intrinsic resistance to HDIs that may be overcome by using combination therapies. As many epigenetic changes are known to be associated with uveal melanoma pathogenesis,^43^ the epigenetic effects of HDIs serve as a promising therapeutic strategy against uveal melanoma. In this study, we demonstrate that combining romidepsin, administered in a manner consistent with the clinical application of the drug, with an mTOR inhibitor increases cell death in uveal melanoma cell lines. RNA Seq analysis suggested that proteins involved in the apoptosis pathway could contribute to this effect and we saw a strong induction of the pro-apoptotic protein Bim in treatments containing romidepsin. Caspase inhibition was also found to abrogate the observed cell death. These data support our hypothesis that the intrinsic apoptotic pathway plays a role in the toxicity observed with the romidepsin/mTOR inhibitor combination.

Previous studies have suggested that HDIs either alone or in combination with targeted therapies could be effective in treating uveal melanoma. Some of the earliest studies with HDIs found romidepsin potently inhibited growth and induced apoptosis in uveal melanoma cell lines. However, it should be noted that the treatment times ranged from 24 to 48 hours, which is not clinically relevant given romidepsin's short half-life and weekly administration schedule.^44^^,^^45^ When treating cells with romidepsin for only 6 hours, a more clinically relevant dosing scheme, we found that romidepsin alone was not capable of inducing strong apoptosis in the uveal melanoma cell lines we tested, except for the 92.1 cell line. Other HDIs, such as valproic acid, panobinostat, vorinostat, tenovin-6, and JSL-1, have also demonstrated efficacy in in vitro studies as well as in vivo studies of uveal melanoma.^46^^–^^48^ In addition to treatment with a single drug, combinations with HDIs have also shown promise in some studies. For example, the combination of quisinostat and the pan-cyclin dependent kinase inhibitor flavopiridol led to apoptotic cell death in uveal melanoma cell lines.^49^ Combination of HDIs with MEK inhibitors was found to thwart escape mechanisms when MEK inhibitors were used as a single therapy.^50^ However, our current study found that mTOR inhibition combined with HDAC inhibition was effective in uveal melanoma cell lines, which may represent another treatment for this disease.

The mTOR pathway is one of the many survival pathways activated by pathogenic variants in the *GNAQ* or *GNA11* genes,^51^ supporting the need for investigations into the efficacy of therapies for uveal melanoma containing mTOR inhibitors. Some in vitro models have suggested that mTOR inhibitors alone may be effective in the treatment of uveal melanoma,^19^ but others have shown that mTOR treatment alone does not result in significant amounts of apoptosis or inhibition of proliferation,^52^^–^^54^ suggesting a need for combination therapies. Combining the mTOR inhibitor rapamycin with the PI3K inhibitor GDC-0941 was found to lead to increased apoptosis in uveal melanoma cell lines as well as patient-derived xenografts.^55^ Interestingly, activation of the PI3K pathway was found to occur in some uveal melanomas lacking *BAP1* pathogenic variants.^56^ An inhibitor of protein kinase C combined with an inhibitor of the mTOR pathway has also been found to be efficacious for uveal melanoma.^53^ A recent study suggested that combining the HDI entinostat with dual human epidermal growth factor 2 and the epidermal growth factor receptor neratininb leads to internalization and degradation of mutant GNA11 and GNAQ in uveal melanoma models.^57^ Interestingly, the lethal effects of this combination were diminished when cells were transfected to express constitutively active mTOR, which led to retention of the anti-apoptotic proteins Bcl-x_L_ and Mcl-1.^57^ Furthermore, the authors of that study found that overexpression of activated mTOR was capable of reducing HDI-induced lethality,^57^ suggesting that the mTOR pathway could cause intrinsic resistance to HDIs. Although our data showed increased expression of Mcl-1 upon HDI treatment, pro-apoptotic proteins seem to overcome this romidepsin-induced expression of Mcl-1 and still promote apoptosis, as seen by Annexin V expression (see Fig. 2) and RNA sequencing analysis (see Fig. 4). Previous studies in our laboratory also revealed increased Mcl-1 expression induced by romidepsin, but only in Ras-mutant cell lines,^10^ which may indicate that the increased expression seen in Mel202 cells is due to the fact that it harbors a *GNAQ* pathogenic variant.

In summary, we demonstrate that the combination of an mTOR inhibitor with a clinically relevant dosage of romidepsin is effective in cell line models of uveal melanoma that harbor pathogenic variants of *GNAQ* or *GNA11*. There is a critical need for developing a more targeted and efficacious therapy for patients with uveal melanoma and our data suggest that inhibiting the mTOR pathway in combination with romidepsin could be an effective treatment option for managing this disease. Furthermore, such studies could provide proof of concept for a tissue-agnostic approach to therapy of other malignancies with pathogenic variants in the guanine nucleotide protein family.

## Supplementary Material

Supplement 1
