# Supplementary material for: Dual Inhibition of Histone Deacetylases and the Mechanistic Target of Rapamycin Promotes Apoptosis in Cell Line Models of Uveal Melanoma
Source: Invest Ophthalmol Vis Sci. 2021 Sep 17;62(12):16. doi: 10.1167/iovs.62.12.16 (PMC8458781; doi:10.1167/iovs.62.12.16)
Supplement: Supplement 1 [file iovs-62-12-16_s001.pdf]

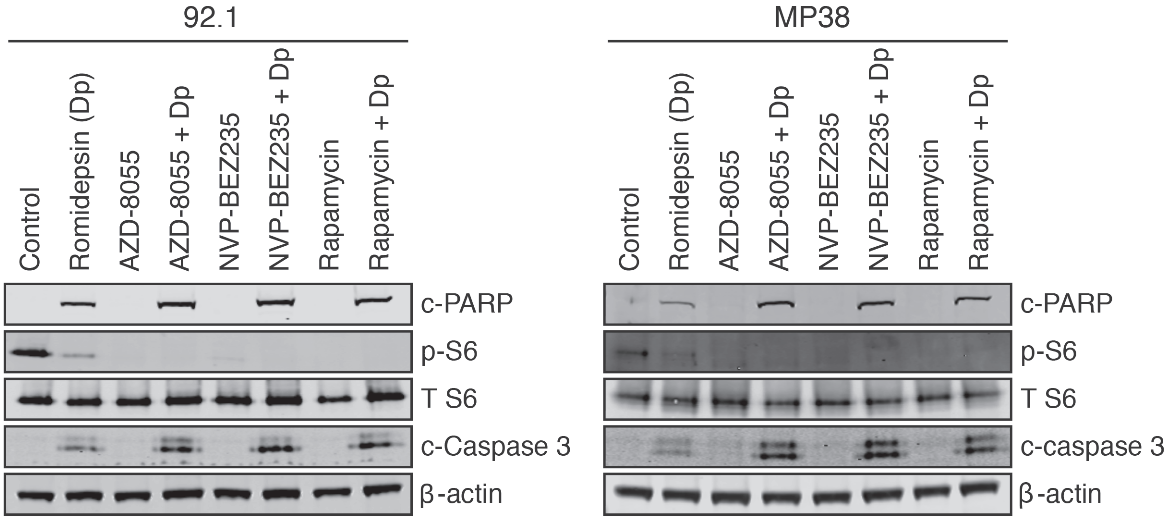

Supplementary Figure 1. Combination of mTOR inhibitors with romidepsin leads to increased PARP and caspase cleavage. 92.1 and MP38 cells were treated as described in the legend to Figure 3. Cell lysates were then prepared, subjected to SDS-PAGE, and transferred to nitrocellulose membranes. Membranes were probed with one or more of the following antibodies: cleaved PARP, cleaved caspase-3, phospho-S6, or S6. β-actin served as a loading control. Each immunoblot was performed in at least 2 independent experiments.

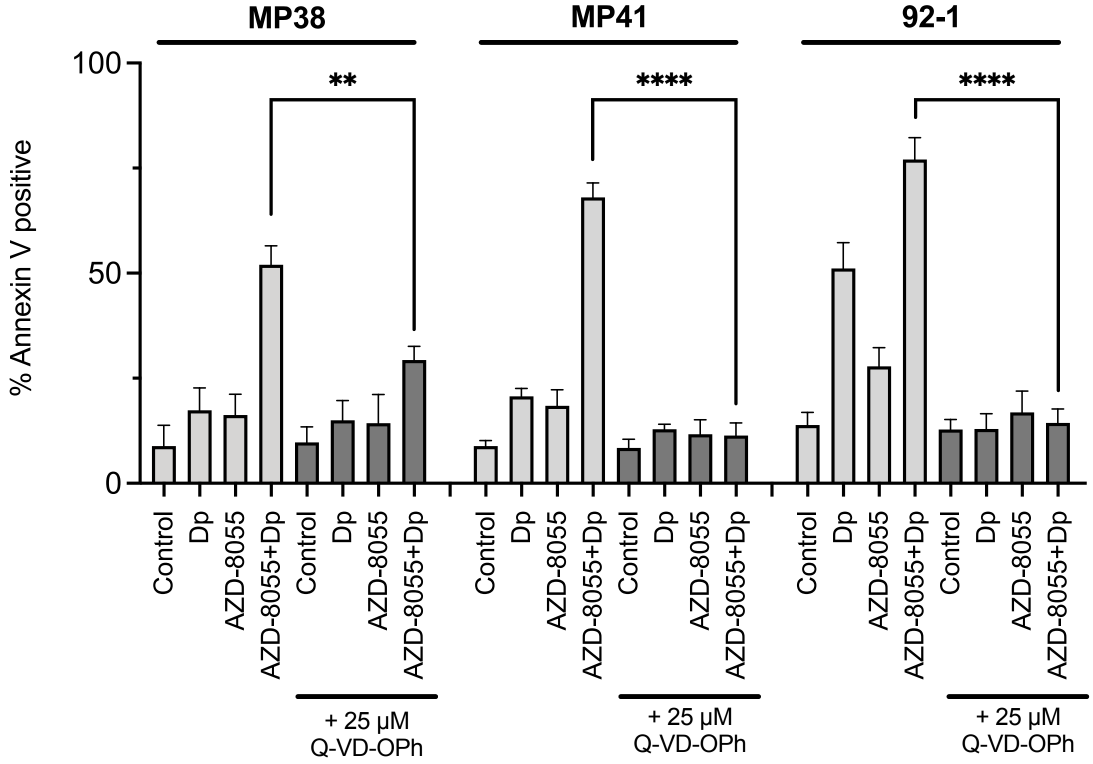

Supplementary Figure 2. Apoptosis induced by combination of romidepsin and AZD-8055 is caspase-dependent. 92.1, MP38 and MP38 cells were treated for 6 h with 25 ng/mL romidepsin alone or in combination with 1 µM AZD-8055 either in the presence or absence of 25 µM of the caspase inhibitor Q-VD-OPh. Cells were then incubated in romidepsin-free media in the absence or presence of AZD-8055 and Q-VD-OPh for an additional 42 h, stained with annexin V and SYTOX green, and analyzed by flow cytometry. Each bar represents the mean of three biological replicates with the standard error of mean indicated by error bars. n = 3.

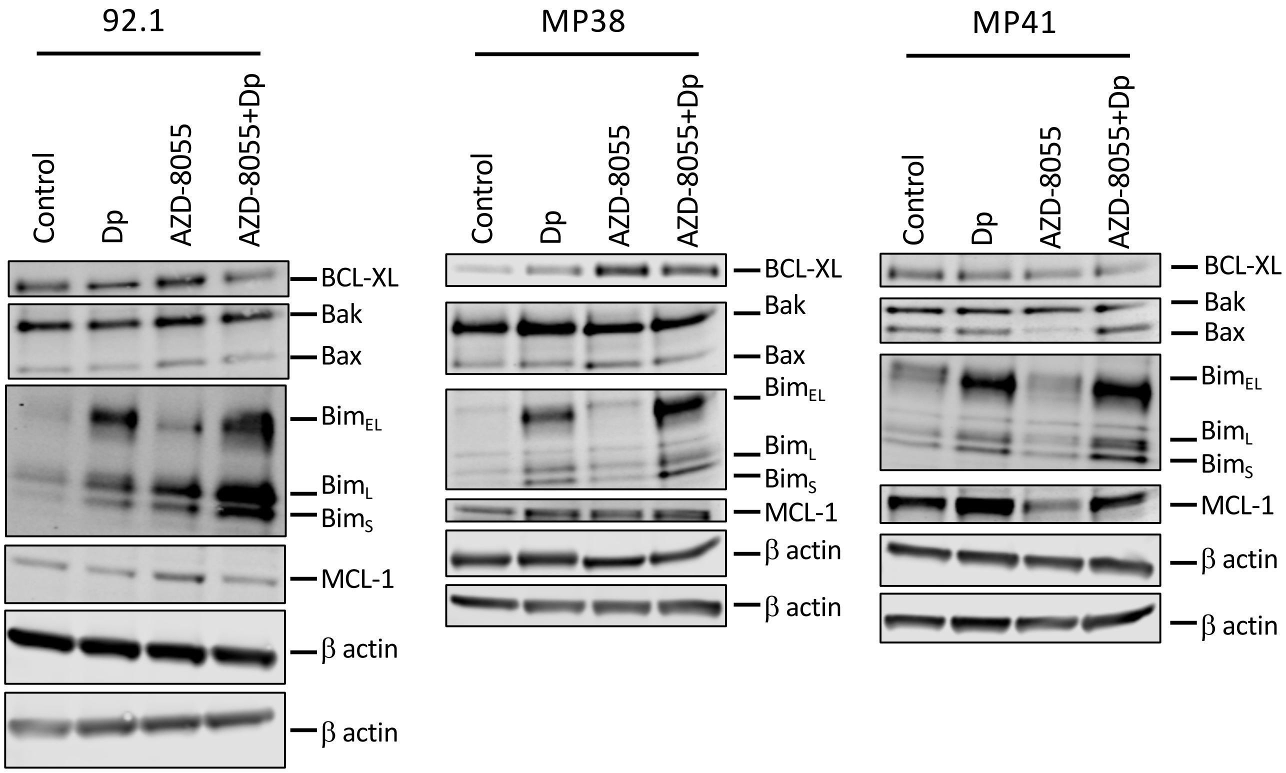

Supplementary Figure 3. Uveal melanoma cell lines treated with romidepsin combined with AZD-8055 show alterations in BCL2 family proteins. 92.1, MP38 and MP41 cells were treated for 6 h with 25 ng/mL romidepsin alone or in combination with 1 µM AZD-8055. Cells were subsequently incubated in romidepsin-free media in the absence or presence of AZD-8055 for an additional 18 h, at which point cells were harvested. Cell lysates were then prepared, subjected to SDS-PAGE, and transferred to nitrocellulose membranes. Membranes were probed with one or more of the following antibodies: Mcl-1, Bim, Bcl-x_L, Bax, or Bak. β-actin served as a loading control. At least 2 independent experiments were performed.

Supplementary Table 1. Characteristics of uveal melanoma cell lines used in this study [1, 2].

**Cell line**	***GNAQ* pathogenic variant**	***GNA11* pathogenic variant**	**BAP1 expression**	***BAP1* pathogenic variant**
92-1	c.626 a > T	-	Yes	-
Mel202	c.629 G > A	-	Yes	-
MP38	c.626 a > T	-	No (loss of splice site)	c.68-9_72del
MP41	-	c.626 a > A/T	Yes	-

References

1. Amirouchene-Angelozzi N, Nemati F, Gentien D, et al. Establishment of novel cell lines recapitulating the genetic landscape of uveal melanoma and preclinical validation of mTOR as a therapeutic target. *Mol Oncol* 2014;8:1508-1520.
2. Griewank KG, Yu X, Khalili J, et al. Genetic and molecular characterization of uveal melanoma cell lines. *Pigment Cell Melanoma Res* 2012;25:182-187.

Supplementary Table 2. STR analysis results for uveal melanoma cell lines

**Locus**	**92.1**	**Mel202**	**MP38**	**MP41**
Amelogenin	X	X	X	X
CSF1PO	10, 11	10, 11	11	9, 10
D13S317	11, 12	11, 13	12	11, 14
D16S539	12	11, 12	9	13
D5S818	9, 11	11, 12	12	10, 12, 13
D7S820	10, 11	11, 12	10, 12	8.3, 10, 10.1, 11
TH01	9, 9.3	6, 7	7, 9	6, 7
TPOX	8, 9	8	8	8, 11
vWA	16	18, 19	14, 17	15, 17, 18

Supplementary Table 3. Compounds used in this study.

Generic name	Target	Tradename	PubChem CID
Romidepsin	HDAC	Istodax	5352062
OTX-015	BRD4	Birabresib	9936746
BVD-523	ERK	Ulixertinib	11719003
AZD-8055	mTOR	-	25262965
GDC-0941	PI3K	Pictilisib	17755052
NVP-BEZ235	mTOR	Dactolisib	11977753
Rapamycin	mTOR	Sirolimus	52846516
Q-VD-OPh	Caspases 1,3,8,9	-	24794416

Supplementary Table 4. Results of annexin-positive cells for each treatment in MP41, Mel202, 92-1, and MP38 cells in Figure 1.

Cell line	Repli-cate	Control	OTX-015	Ulixer-tinib	GDC-0941	AZD-8055	GDC +OTX	GDC + Uli	AZD +OTX	AZD +Uli	DP	OTX +DP	Uli +DP	GDC +DP	AZD +DP	GDC +OTX +DP	GDC +Uli +DP	AZD +OTX +DP	AZD +Uli +DP
**MP41**																			
	R1	8.11	18.59	10.99	9.52	19.74	25.7	24.32	28.6	27.31	34	54.7	55.2	58.6	63	68.8	68.7	64.7	72.1
	R2	4.24	27.9	12.12	8.17	15.98	25.3	16.8	43.6	17.63	23.6	35.9	41.4	53.5	59.4	55.5	66.5	66.8	69.1
	R3	9.03	19.38	12.05	10.59	21.48	25.33	24.5	36.2	27.19	25	50.1	48.9	50.1	67.4	70.9	74.9	70.4	74
	**Mean ± SD**	**7.13 ± 2.54**	**21.96 ± 5.16**	**11.72 ± 0.63**	**9.43 ± 1.21**	**19.07 ± 2.81**	**25.44 ± 0.22**	**21.87 ± 4.39**	**36.13 ± 7.50**	**24.04 ± 5.55**	**27.53 ± 5.64**	**46.90 ± 9.80**	**48.50 ± 6.91**	**54.07 ± 4.28**	**63.27 ± 4.01**	**65.07 ± 8.35**	**70.03 ± 4.36**	**67.30 ± 2.88**	**71.73 ± 2.47**
**Mel202**																			
	R1	6.98	9.91	15.04	8.94	36.7	17.55	27.71	50.6	52	14.45	27.7	33	44.9	72.4	27.24	54.4	73.8	74.2
	R2	7.26	14.72	20.67	13.14	36.9	19.85	41.59	75.9	67	19.53	34.2	31.8	48.9	82.5	57.9	66.1	91.2	86.8
	R3	8.58	12.03	14.71	9.25	35.35	19.75	33.92	55.79	61.36	15.11	31.95	33.01	34.8	75.31	55.98	61.6	79.24	76.81
	**Mean ± SD**	**7.61 ± 0.85**	**12.22 ± 2.41**	**16.81 ± 3.35**	**10.44 ± 2.34**	**36.32 ± 0.84**	**19.05 ± 1.30**	**34.41 ± 6.95**	**60.76 ± 13.36**	**60.12 ± 7.58**	**16.36 ± 2.76**	**31.28 ± 3.30**	**32.60 ± 0.70**	**42.87 ± 7.27**	**76.74 ± 5.20**	**47.04 ± 17.17**	**60.70 ± 5.90**	**81.41 ± 8.90**	**79.27 ± 6.65**
**92.1**																			
	R1	4.83	8.38	6.69	8.54	13.98	27.32	10.68	26.42	12.34	31.94	43	37.99	42.49	38.7	49.77	44.9	52.54	41.1
	R2	6.17	12.87	10.8	11.07	23.9	20.31	17.84	52.4	26.5	55.8	69.6	67.5	63.8	62.6	57.1	68.8	63.2	58.5
	R3	5.78	10.77	12.36	7.76	20.48	20.93	14.11	49.1	22.57	57	62.8	61.6	65.7	73.1	72.6	74.57	81.6	74
	**Mean ± SD**	**5.59 ± 0.69**	**10.67 ± 2.25**	**9.95 ± 2.93**	**9.12 ± 1.73**	**19.45 ± 5.04**	**22.85 ± 3.88**	**14.21 ± 3.58**	**42.64 ± 14.14**	**20.47 ± 7.31**	**48.25 ± 14.13**	**58.47 ± 13.82**	**55.70 ± 15.62**	**57.33 ± 12.89**	**58.13 ± 17.63**	**59.82 ± 11.66**	**62.76 ± 15.73**	**65.78 ± 14.70**	**57.87 ± 16.46**
**MP38**																			
	R1	8.87	22.06	21.6	15.86	15.35	32.02	27.4	40.75	43.15	31.84	67.11	53.48	52.86	61.01	81.17	77.51	71.11	67.17
	R2	6.15	21.8	14.31	9.03	11.5	39.7	20.86	36.9	37.08	32.5	67.1	46.4	50.91	48.44	76.88	69.08	71.68	56.35
	R3	4.36	16.84	8.81	6.46	16.57	36.49	18.72	42.31	33.79	27	62.26	38.82	52.59	58.22	71.07	66.82	73.66	60.1
	**Mean ± SD**	**6.46 ± 2.27**	**20.23 ± 2.94**	**14.91 ± 6.42**	**10.45 ± 4.86**	**14.47 ± 2.65**	**36.07 ± 3.86**	**22.33 ± 4.52**	**39.99 ± 2.78**	**38.01 ± 4.75**	**30.45 ± 3.00**	**65.49 ± 2.80**	**46.23 ± 7.33**	**52.12 ± 1.06**	**55.89 ± 6.60**	**76.37 ± 5.07**	**71.14 ± 5.63**	**72.15 ± 1.34**	**61.21 ± 5.49**
